# Expression, purification, and crystallization of *Schizosaccharomyces pombe* eIF2B

**DOI:** 10.1007/s10969-016-9203-3

**Published:** 2016-03-29

**Authors:** Kazuhiro Kashiwagi, Tomoaki Shigeta, Hiroaki Imataka, Takuhiro Ito, Shigeyuki Yokoyama

**Affiliations:** Graduate School of Science, The University of Tokyo, 7-3-1 Hongo, Bunkyo-ku, Tokyo, 113-0033 Japan; RIKEN Systems and Structural Biology Center, 1-7-22 Suehiro-cho, Tsurumi-ku, Yokohama, 230-0045 Japan; RIKEN Center for Life Science Technologies, 1-7-22 Suehiro-cho, Tsurumi-ku, Yokohama, 230-0045 Japan; Graduate School of Engineering, University of Hyogo, Himeji, 671-2280 Japan; RIKEN Structural Biology Laboratory, 1-7-22 Suehiro-cho, Tsurumi-ku, Yokohama, 230-0045 Japan

**Keywords:** eIF2B, Eukaryotic translation initiation factor, Guanine-nucleotide exchange factor

## Abstract

Tight control of protein synthesis is necessary for cells to respond and adapt to environmental changes rapidly. Eukaryotic translation initiation factor (eIF) 2B, the guanine nucleotide exchange factor for eIF2, is a key target of translation control at the initiation step. The nucleotide exchange activity of eIF2B is inhibited by the stress-induced phosphorylation of eIF2. As a result, the level of active GTP-bound eIF2 is lowered, and protein synthesis is attenuated. eIF2B is a large multi-subunit complex composed of five different subunits, and all five of the subunits are the gene products responsible for the neurodegenerative disease, leukoencephalopathy with vanishing white matter. However, the overall structure of eIF2B has remained unresolved, due to the difficulty in preparing a sufficient amount of the eIF2B complex. To overcome this problem, we established the recombinant expression and purification method for eIF2B from the fission yeast *Schizosaccharomyces pombe*. All five of the eIF2B subunits were co-expressed and reconstructed into the complex in *Escherichia coli* cells. The complex was successfully purified with a high yield. This recombinant eIF2B complex contains each subunit in an equimolar ratio, and the size exclusion chromatography analysis suggests it forms a heterodecamer, consistent with recent reports. This eIF2B increased protein synthesis in the reconstituted in vitro human translation system. In addition, disease-linked mutations led to subunit dissociation. Furthermore, we crystallized this functional recombinant eIF2B, and the crystals diffracted to 3.0 Å resolution.

## Introduction

Eukaryotic translation initiation factor (eIF) 2B catalyzes guanine nucleotide exchange on eIF2, which delivers an initiator methionyl-tRNA to the ribosome in a GTP-dependent manner [1, 2]. As eIF2 has stronger affinity for GDP than GTP [3], the conversion of inactive eIF2·GDP into active eIF2·GTP requires the activity of eIF2B. Therefore, the inhibition of eIF2B activity represses most of the translation initiation events, and diverse stressors are known to induce the inhibition of eIF2B [4]. Among such mechanisms, the inhibition by the phosphorylated eIF2 has been well described. eIF2 kinases sense various stressors, and phosphorylate eIF2. The phosphorylated eIF2 inhibits the nucleotide exchange activity of eIF2B, by acting as a competitive inhibitor against the unphosphorylated eIF2 [5]. Thereby, the cellular level of eIF2·GTP is lowered and translation is reduced. eIF2B is a protein complex consisting of the α, β, γ, δ, and ε subunits. The phosphorylation of eIF2 is recognized by the regulatory subcomplex, composed of the α, β, and δ subunits [6], while the nucleotide exchange on eIF2 is catalyzed by the catalytic subcomplex, composed of the γ and ε subunits [7].

eIF2B had long been considered to be a heteropentameric protein. However, recent studies suggested that eIF2B is a heterodecamer, and there are still some conflicts among these studies about the configuration of the subunits [8–11]. This obscurity of the fundamental structure of eIF2B is partly due to the difficulty in the preparation of homogeneous eIF2B protein. In eukaryotic cells, eIF2B is one of the least abundant proteins among the eukaryotic initiation factors [12], and its activity is modulated by various posttranslational modifications. Therefore, the establishment of a recombinant method to obtain a large amount of homogeneous eIF2B, for biochemical and structural studies, has long been awaited. In addition, the neurodegenerative disease, leukoencephalopathy with vanishing white matter or childhood ataxia with central nervous system hypomyelinization (VWM/CACH), is caused by mutations in the eIF2B subunits [13]. The mutations reportedly affect the functions of eIF2B in various manners. However, the lack of information about the three-dimensional structure of eIF2B has hindered the comprehensive understanding of the pathogenesis of VWM/CACH disease.

Here we report the recombinant expression, purification, and crystallization of eIF2B, from the fission yeast *Schizosaccharomyces pombe*.

## Materials and methods

### Expression and purification of *S. pombe* eIF2B

**Fig. 1:**
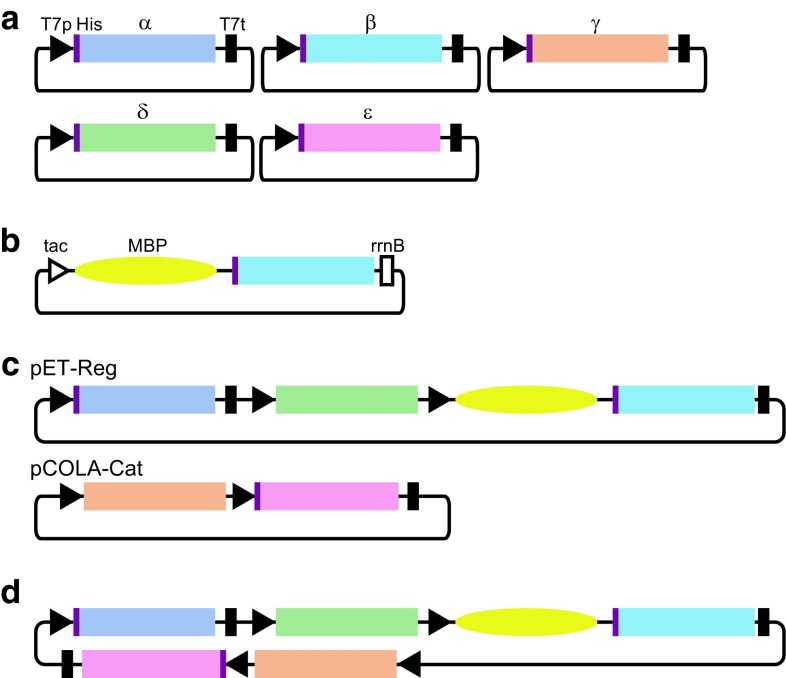
The expression constructs used in this study. **a** The vectors for the expression of each subunit. The positions of the T7 promoter, the T7 terminator, and the His_6_-tag are shown by *black triangles* (T7p), *black bars* (T7t), and *purple bars* (His), respectively. **b** The expression vector for the MBP-His_6_-fused β subunit. The positions of the tac promoter, the rrnB terminator, and the MBP-tag are shown by the *open triangle* (tac), the *open bar* (rrnB), and the *yellow oval* (MBP), respectively. **c** The expression vectors for the co-expression. The pET-Reg vector carries the sequences for His_6_-tag fused eIF2Bα, eIF2Bδ, and MBP-His_6_-tag fused eIF2Bβ. The pCOLA-Cat vector carries the sequences for eIF2Bγ and His_6_-tag fused eIF2Bε. **d** The expression vector for the co-expression of all five subunits. All tag sequences fused to the eIF2B subunits are followed by the HRV 3C protease recognition sequence

DNA fragments, each encoding one subunit of eIF2B, were amplified by polymerase chain reaction (PCR) from an *S. pombe* cDNA library. The fragments were cloned into the NdeI/NotI sites (for the α, β, δ, and ε subunits) or the BamHI/NotI sites (for the γ subunit) of a derivative of the vector pET-28c (Novagen), in which the recognition sequence of thrombin had been replaced with that of the HRV 3C protease (Fig. 1a). For the expression of the MBP-His_6_-fused β subunit, the fragment encoding the β subunit was cloned into the EcoRI/SalI sites of a derivative of pMAL-c2X (New England Biolabs), in which the Factor Xa recognition sequence was replaced with the His_6_ tag and the HRV 3C protease recognition sequence (pMAL-2Bβ; Fig. 1b). For the construction of the plasmid to co-express the regulatory subunits, the fragment encoding the δ subunit was cloned into the NcoI/NotI sites of pETDuet-1 (Novagen; pETDuet-1-2Bδ). The fragment encoding the MBP-His_6_-fused β subunit was amplified by PCR from pMAL-2Bβ, and cloned into the NdeI/XhoI sites of pETDuet-1-2Bδ (pETDuet-1-2Bδ-MBP-His_6_-2Bβ). The fragment encoding the His_6_-fused α subunit was amplified with the T7 promoter and terminator sequences, and cloned into the SgrAI/SphI sites of pETDuet-1-2Bδ-MBP-His_6_-2Bβ (pET-Reg; Fig. 1c). Each of the mutations corresponding to human VWM/CACH mutations was introduced into this plasmid using a PrimeSTAR Mutagenesis Basal Kit (Takara). For the construction of the plasmid to co-express the catalytic subunits, the DNA fragment encoding the ε subunit was also amplified by PCR from the cDNA library, and cloned into the EcoRI/NotI sites of pET-47b (Novagen). The fragment encoding the His_6_-fused ε subunit was amplified and cloned into the NdeI/XhoI sites of pCOLADuet-1 (Novagen; pCOLADuet-1-His_6_-2Bε). The fragment encoding the γ subunit, amplified from the cDNA library, was cloned into the NcoI/HindIII sites of pCOLADuet-1-His_6_-2Bε (pCOLA-Cat; Fig. 1c). For the construction of the plasmid to co-express all five of the subunits, the recognition sequences for SpeI and FseI were inserted upstream of the SgrAI site of pET-Reg, using a PrimeSTAR Mutagenesis Basal Kit, and then the 2Bγ-His_6_-2Bε sequence was amplified with the T7 promoter and terminator sequences from pCOLA-Cat, and cloned into the SpeI/FseI sites (Fig. 1d).

For the expression and purification of eIF2B, the *E. coli* strain Rosetta2(DE3) (Novagen) was co-transformed with pET-Reg and pCOLA-Cat, and grown in LB medium supplemented with 0.2 % glucose and appropriate antibiotics at 310 K. After the addition of 0.3 mM isopropyl-β-d-thiogalactopyranoside (IPTG) when the culture reached an OD_600_ of 0.5, the cells were grown at 291 K overnight. The cells were harvested, and lysed by sonication in buffer A [20 mM HEPES–KOH buffer (pH 7.5) containing 150 mM KCl, 10 % (v/v) glycerol, 1 mM dithiothreitol (DTT), and protease inhibitors (Roche)] at 277 K. All further purification steps were also performed at 277 K, unless otherwise noted. After the removal of insoluble materials by centrifugation, the supernatant was applied to Amylose Resin (New England Biolabs), and eluted with buffer A supplemented with 10 mM maltose. The protein was further purified by chromatography on a HiTrap Heparin column (GE Healthcare), and eluted with a linear gradient of 0.15–1 M KCl in buffer B [20 mM HEPES–KOH buffer (pH 7.5) containing 5 % (v/v) glycerol and 1 mM DTT]. The fraction containing all five of the subunits was collected, and dialyzed overnight with the HRV 3C protease against buffer C [20 mM HEPES–KOH buffer (pH 7.5) containing 150 mM KCl, 5 % (v/v) glycerol, and 1 mM DTT]. After supplementation with 20 mM imidazole, the sample was passed through a Ni-Sepharose column (GE Healthcare), and then purified on a Sephacryl S-300 column (GE Healthcare) in buffer C. For the estimation of the molecular masses, the peak fraction and a Gel Filtration Calibration Kit HMW (GE Healthcare) were applied to a Superose 6 column (GE Healthcare), equilibrated with buffer C.

### Luciferase reporter assay

Human eIF2 and eIF2B were prepared as described previously [14]. The translation of *Renilla* luciferase (Rluc) in a reconstituted human translation system and the measurement of luciferase activity were performed as described previously, with the following modifications [15]. The Rluc mRNA was transcribed from the template plasmid (pUC-T7-HCV-HA-Rluc-FLAG), and purified in advance. This mRNA was included in the reconstituted system, instead of the plasmid and T7 RNA polymerase. In addition, the concentration of GTP was reduced to 0.21 mM. The system was supplemented with eIF2 and eIF2B, at final concentrations of 0.24 and 1.6 μM, respectively. The luciferase activity was measured as described previously [15].

### Crystallization and data collection

The purified eIF2B was concentrated to 6 mg/ml. Crystals were grown at 293 K on MRC Maxi 48-Well Crystallization Plates (Swissci), by the sitting drop vapor diffusion method. The mixture of 2 μl of the protein solution and 2 μl of the reservoir solution [50.4 mM NaH_2_PO_4_, 49.6 mM K_2_HPO_4_, 200 mM NaCl, and 12 % polyethylene glycol monomethyl ether (PEGMME) 2000] was equilibrated against 120 μl of the reservoir solution. Crystals appeared in about 2 days, and grew to an approximate size of 250 × 100 × 50 μm within 5 days. The crystals were harvested at 293 K, and first immersed in a solution containing 50.4 mM NaH_2_PO_4_, 49.6 mM K_2_HPO_4_, 180 mM NaCl, 12 % PEGMME 2000, and 5 % (v/v) glycerol. For cryoprotection, the crystals were serially transferred into solutions supplemented with 2 % (v/v) glycerol and 2 % (v/v) dimethyl sulfoxide (DMSO). After five steps of transfer [the final concentrations were 15 % (v/v) for glycerol and 10 % (v/v) for DMSO], the crystals were flash-cooled by plunging them into liquid nitrogen. The conditions for the crystallization and the data collection were optimized, using the beamlines at the Photon Factory (Ibaraki, Japan) and SPring-8 (Hyogo, Japan). Initial X-ray diffraction data were collected at the beamline BL32XU, and the final data were collected at BL41XU at SPring-8. The data set containing 300 frames was collected at 100 K with an oscillation angle of 0.5° per frame, using a MAR225HE detector. The data set was processed with XDS [16]. The data collection statistics are summarized in Table 1.

**Table 1:** X-ray data collection and processing

	eIF2B
Crystal parameters	
Space group	*P*2_1_2_1_2_1_
Cell dimensions: a, b, c (Å)	144.5, 209.2, 223.5
Data collection	
Wavelength (Å)	1.0000
Temperature (K)	100
Detector	MAR225HE
Crystal-detector distance (mm)	320
Rotation per image (°)	0.5
Total rotation range (°)	150
Exposure time per image (s)	1
Mosaicity (°)	0.097
Resolution (Å)	49.29–2.99 (3.17–2.99)
R_meas_ (%)	15.3 (170)
Total No. of reflections	860,670 (134,307)
No. of unique reflections	136,112 (21,332)
Mean redundancy	6.3 (6.3)
Overall completeness (%)	99.6 (97.9)
Mean I/σ	13.3 (1.3)
CC_1/2_	0.998 (0.524)

Entries in parentheses represent data from the limiting resolution shell. Data collection and refinement statistics were determined with *XDS* [16]

## Results and discussion

**Fig. 2:**
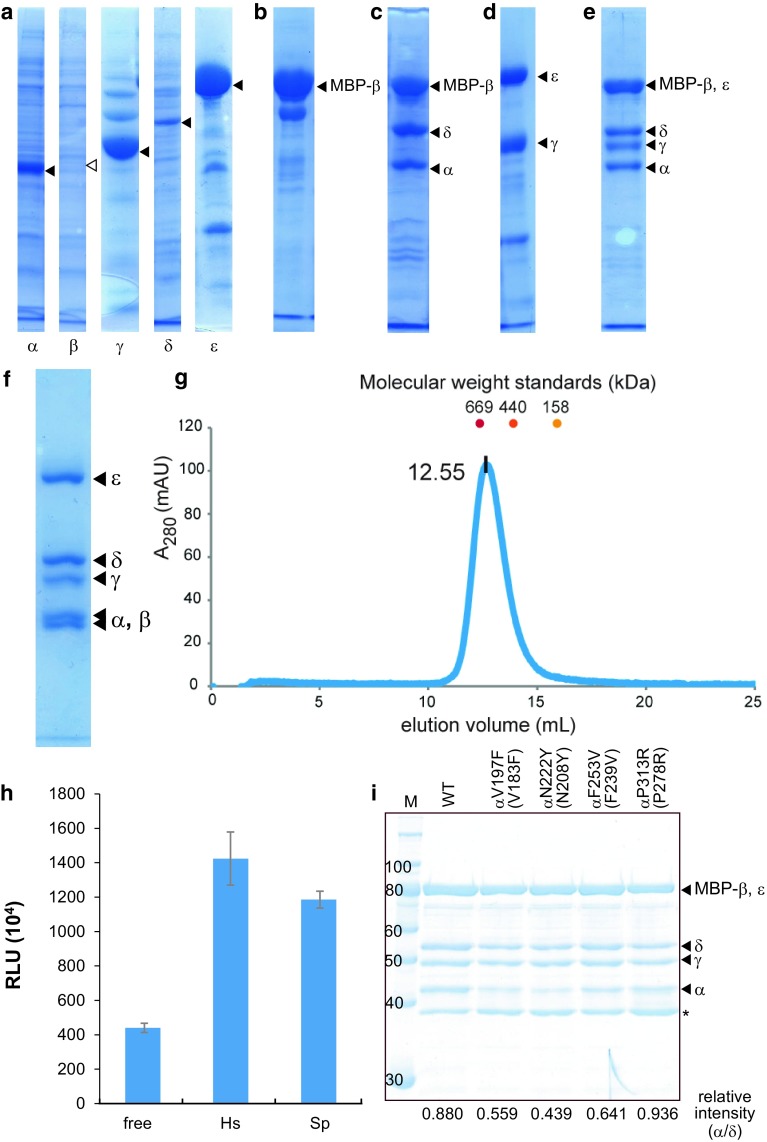
Analyses of recombinant eIF2B. **a**–**e** The eIF2B subunits from various constructs were resolved by SDS-PAGE, and stained with Coomassie Brilliant Blue. The expression of each subunit from the vectors in Fig. 1a (**a**), MBP-His_6_-fused β subunit (**b**), the co-expression from pET-Reg (**c**), pCOLA-Cat (**d**), and the co-expression of five subunits from pET-Reg and pCOLA-Cat (**e**). The acrylamide concentrations of the gels are 10 (**a**–**c**, **e**) and 12 % (**d**). **f** Purified eIF2B complex. **g** The SEC analysis of the recombinant eIF2B. The chromatogram of the absorbance at 280 nm is shown (*left*). The elution volumes of the molecular weight standards are indicated with dots [thyroglobulin (669 kDa), ferritin (440 kDa) and aldolase (158 kDa)]. **h** The assay for the eIF2B activity in a reconstituted human translation system. The activity of *Renilla* luciferase synthesized by HCV IRES-directed translation was measured. Human eIF2 only (free), human eIF2 and eIF2B (Hs), and human eIF2 and *S. pombe* eIF2B (Sp) were respectively included in the reconstituted translation system. The error bars represent standard deviations from triplicate analyses. **i** The assay for the eIF2B complex formation. The wild type and four VWM/CACH-linked mutants of eIF2B were purified and resolved by SDS-PAGE. The MBP tag fused to the β subunit was used for affinity purification. The band intensities were quantified, and the relative intensities of the α subunit to the δ subunit, which forms a stable heterodimer with the β subunit [11], are shown *below the lanes*

In order to obtain a large amount of recombinant *S. pombe* eIF2B, we first tried to reconstruct the eIF2B complex in vitro, from the individually prepared subunits. However, this trial was unsuccessful because of the insolubility of the subunits, especially the β subunit (Fig. 2a). The solubility of the β subunit was partially improved by using the MBP fusion construct (Fig. 2b), but the β subunit was still unstable and prone to precipitation. Next, we aimed to stabilize the MBP-fused β subunit by co-expression with the α and δ subunits. We cloned these three subunits into a single plasmid (“pET-Reg” in Fig. 1c), and co-expressed the three regulatory subunits. This successfully reconstructed the regulatory subcomplex (Fig. 2c). However, the purification of this subcomplex was unsuccessful, because the α subunit dissociated under high salt conditions.

The DNA fragments encoding the catalytic γ and ε subunits were cloned into pCOLADuet-1 (“pCOLA-Cat” in Fig. 1c). pCOLA-Cat expresses the non-tagged γ subunit and the N-terminally His_6_-fused ε subunit, and these subunits formed the catalytic subcomplex (Fig. 2d). The co-expression from these two constructed plasmids resulted in the successful expression of all five subunits, and the eIF2B complex was reconstructed in *E. coli* cells (Fig. 2e). We further concatenated these constructs into a single plasmid, but obtained reduced expression levels. Thus, we established the method for the recombinant expression of eIF2B by the co-expression of the five subunits from two plasmids. The N-terminal tags fused to the subunits are all cleavable by the HRV 3C protease.

The recombinant eIF2B protein expressed by this method was purified through three affinity chromatographies and size exclusion chromatography. The SDS-PAGE analysis of the purified eIF2B complex confirmed that it contains each subunit in an equimolar ratio (Fig. 2f). In the size exclusion chromatography analysis, the eIF2B protein eluted in between thyroglobulin (669 kDa) and ferritin (440 kDa; Fig. 2g). This suggested that the recombinant eIF2B protein is a heterodecamer (518 kDa) containing two copies each of the five subunits, consistent with recent reports [8–11]. The typical yield of this recombinant eIF2B protein was 2–3 mg from 1 liter of an overnight LB culture.

To examine whether this recombinant *S. pombe* eIF2B is functional, we performed HCV IRES-directed translation of *Renilla* luciferase in a reconstituted human translation system [15] with eIF2 at a low GTP concentration. The supplementation of this system with human eIF2B greatly increased the synthesis of luciferase (Fig. 2h). The supplementation with the recombinant *S. pombe* eIF2B, instead of human eIF2B, similarly enhanced translation, showing that this *S. pombe* eIF2B possesses comparable activity to human eIF2B (Fig. 2h). Furthermore, the introduction of mutations that correspond to human VWM/CACH mutations into the α subunit of the recombinant *S. pombe* eIF2B resulted in a mild reduction in the amount of the α-subunit-containing eIF2B complex for three of the four mutants tested (Fig. 2i). A defect in the association of the α subunits was also reported for the mutation-containing eIF2B purified from human cells [17], suggesting that *S. pombe* eIF2B serves as an alternative target to study the effects of human VWM/CACH mutations.

**Fig. 3:**
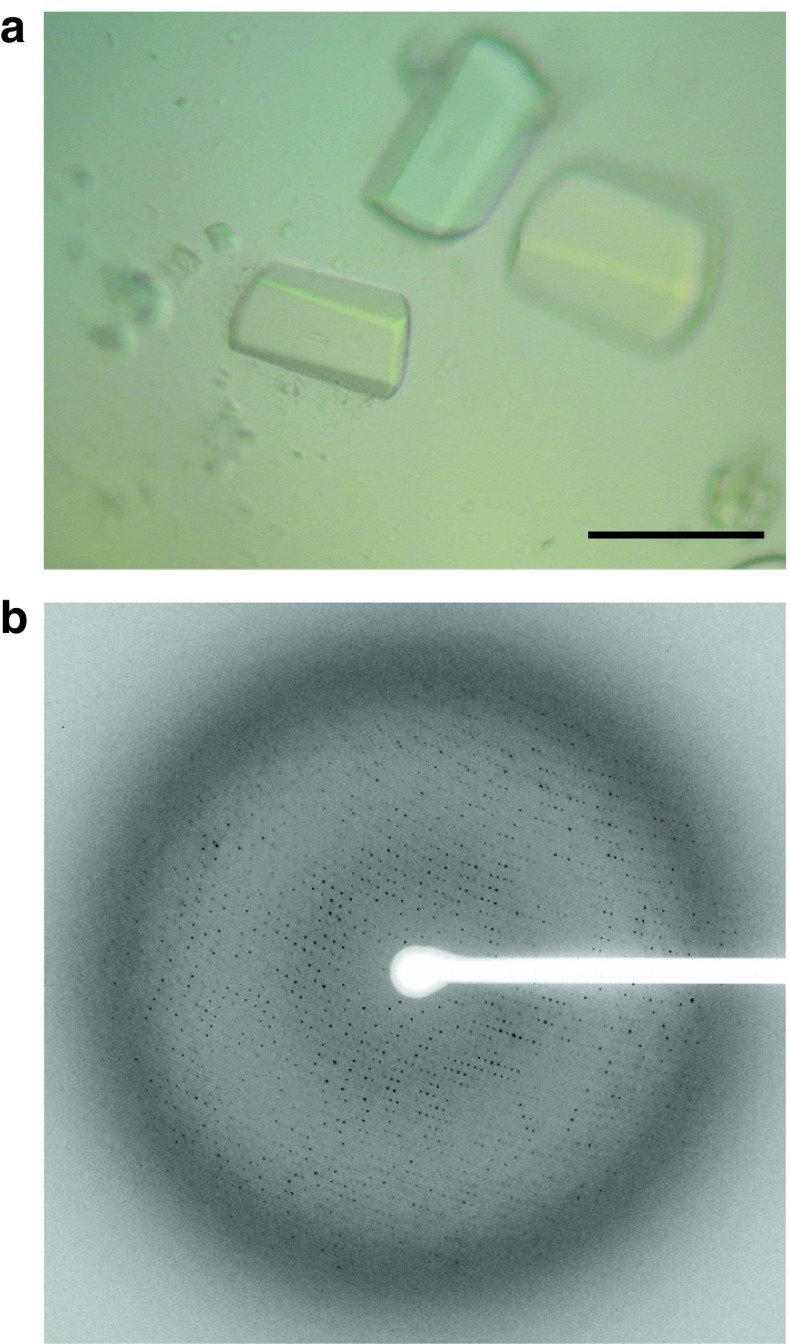
Crystals of *S. pombe* eIF2B and the diffraction pattern. **a** Crystals of the purified recombinant eIF2B. The *black bar* indicates 200 μm. **b** A diffraction image from a crystal of eIF2B

The crystallization screening of the recombinant *S. pombe* eIF2B protein was performed by the sitting drop vapor diffusion method. An initial hit was achieved with No. 6 in the Protein Complex Suite screening kit (Qiagen). Further refinement of the crystallization conditions allowed us to obtain large crystals (Fig. 3a), and they diffracted well when cryoprotected with glycerol or DMSO. For the cryoprotection, the crystals needed to be transferred serially into solutions to increase the cryoprotectant concentrations gradually, to prevent the crystals from cracking. The best diffraction was obtained from the crystals cryoprotected with a mixture of glycerol and DMSO at a ratio of 3:2. The crystals belonged to the space group *P*2_1_2_1_2_1_, with unit cell parameters *a* = 144.5, *b* = 209.2, *c* = 223.5 (Å), and the X-ray diffraction data up to 3.0 Å resolution were collected (Fig. 3b; Table 1). Attempts to collect datasets from a selenomethionine derivative of the recombinant eIF2B, for phasing by the single-wavelength anomalous dispersion method, were successful, and the crystal structure of eIF2B has been determined [18].

Our method for the recombinant production of the eIF2B protein enables the homogeneous preparation of mutation-containing eIF2B proteins, which sometimes affect growth and viability when expressed in eukaryotic cells, and the introduction of protein engineering techniques, such as the site-specific incorporation of non-natural amino acids. This method will contribute to the understanding of the detailed mechanisms of nucleotide exchange and its inhibition, and the pathogenesis of VWM/CACH disease.
